# Outcome of intracerebral hemorrhage associated with different oral anticoagulants

**DOI:** 10.1212/WNL.0000000000003886

**Published:** 2017-05-02

**Authors:** Duncan Wilson, David J. Seiffge, Christopher Traenka, Ghazala Basir, Jan C. Purrucker, Timolaos Rizos, Oluwaseun A. Sobowale, Hanne Sallinen, Shin-Joe Yeh, Teddy Y. Wu, Marc Ferrigno, Rik Houben, Floris H.B.M. Schreuder, Luke A. Perry, Jun Tanaka, Marion Boulanger, Rustam Al-Shahi Salman, Hans R. Jäger, Gareth Ambler, Clare Shakeshaft, Yusuke Yakushiji, Philip M.C. Choi, Julie Staals, Charlotte Cordonnier, Jiann-Shing Jeng, Roland Veltkamp, Dar Dowlatshahi, Stefan T. Engelter, Adrian R. Parry-Jones, Atte Meretoja, David J. Werring, And the CROMIS-2 collaborators

**Affiliations:** From the Stroke Research Center (D.W., C.S., D.J.W.) and Neuroradiological Academic Unit (H.R.J.), Department of Brain Repair and Rehabilitation, Institute of Neurology, and Department of Statistical Science (G.A.), UCL, London, UK; Stroke Center and Neurology (D.J.S., C.T., S.T.E.), University Hospital Basel, University of Basel, Switzerland; Ottawa Hospital Research Institute and University of Ottawa (G.B., D.D.), Canada; Department of Neurology (J.C.P., T.R.), Heidelberg University Hospital, Germany; Manchester Academic Health Sciences Center (O.A.S., A.R.P.-J.), Salford Royal NHS Foundation Trust, UK; Department of Neurology (H.S., A.M.), Helsinki University Hospital, Finland; Stroke Center & Department of Neurology (S.-J.Y., J.-S.J.), Department of Neurology, National Taiwan University Hospital, Taipei; Department of Medicine and neurology at the Royal Melbourne Hospital (T.Y.W., A.M.), University of Melbourne, Parkville, Australia; U1171–Degenerative & Vascular Cognitive Disorders (M.F., C.C.), Univ Lille, Inserm, CHU Lille, France; Department of Neurology (R.H., F.H.B.M.S., J.S.), Maastricht University Medical Center, the Netherlands; Department of Neurosciences (J.A.P., P.M.C.C.), Eastern Health, Melbourne, Australia; Division of Neurology, Department of Internal Medicine (J.T., Y.Y.), Saga University Faculty of Medicine, Japan; Division of Clinical Neurosciences (M.B., R.A.-S.S.), Center for Clinical Brain Sciences, School of Clinical Sciences, University of Edinburgh; Department of Stroke Medicine, Division of Brain Sciences (R.V.), Imperial College London, UK; and Neurorehabilitation Unit (S.T.E.), University of Basel and University Center for Medicine of Aging, Felix Platter Hospital, Switzerland.

## Abstract

**Objective:**

In an international collaborative multicenter pooled analysis, we compared mortality, functional outcome, intracerebral hemorrhage (ICH) volume, and hematoma expansion (HE) between non–vitamin K antagonist oral anticoagulation–related ICH (NOAC-ICH) and vitamin K antagonist–associated ICH (VKA-ICH).

**Methods:**

We compared all-cause mortality within 90 days for NOAC-ICH and VKA-ICH using a Cox proportional hazards model adjusted for age; sex; baseline Glasgow Coma Scale score, ICH location, and log volume; intraventricular hemorrhage volume; and intracranial surgery. We addressed heterogeneity using a shared frailty term. Good functional outcome was defined as discharge modified Rankin Scale score ≤2 and investigated in multivariable logistic regression. ICH volume was measured by ABC/2 or a semiautomated planimetric method. HE was defined as an ICH volume increase >33% or >6 mL from baseline within 72 hours.

**Results:**

We included 500 patients (97 NOAC-ICH and 403 VKA-ICH). Median baseline ICH volume was 14.4 mL (interquartile range [IQR] 3.6–38.4) for NOAC-ICH vs 10.6 mL (IQR 4.0–27.9) for VKA-ICH (*p* = 0.78). We did not find any difference between NOAC-ICH and VKA-ICH for all-cause mortality within 90 days (33% for NOAC-ICH vs 31% for VKA-ICH [*p* = 0.64]; adjusted Cox hazard ratio (for NOAC-ICH vs VKA-ICH) 0.93 [95% confidence interval (CI) 0.52–1.64] [*p* = 0.79]), the rate of HE (NOAC-ICH n = 29/48 [40%] vs VKA-ICH n = 93/140 [34%] [*p* = 0.45]), or functional outcome at hospital discharge (NOAC-ICH vs VKA-ICH odds ratio 0.47; 95% CI 0.18–1.19 [*p* = 0.11]).

**Conclusions:**

In our international collaborative multicenter pooled analysis, baseline ICH volume, hematoma expansion, 90-day mortality, and functional outcome were similar following NOAC-ICH and VKA-ICH.

Randomized trials in patients with atrial fibrillation (AF) show that direct (non–vitamin K antagonist [VKA]) oral anticoagulants (NOACs) have about half the incidence of intracerebral hemorrhage (ICH) compared to VKA but with a similar efficacy in preventing ischemic stroke.^1^ Nevertheless, there is concern that without wide access to specific reversal agents at the time of this study, NOAC-associated ICH (NOAC-ICH) might be larger, with a higher risk of hematoma expansion (HE) and worse outcome, than VKA-associated ICH (VKA-ICH), for which reversal strategies are established.^2–4^

There are few data on the clinical and radiologic characteristics or the functional outcome of NOAC-ICH to guide clinicians. A multicenter prospective study of 61 patients with NOAC-ICH reported 28% mortality at 90 days, but with no comparison to VKA-ICH.^5^ Subanalyses of the RE-LY, ARISTOTLE, and ROCKET-AF trials^6–8^ suggest similar mortality for VKA-ICH and NOAC-ICH. However, a single-center study from Japan (NOAC-ICH, n = 5) and a small multicenter study from the United Kingdom (NOAC-ICH, n = 11) both found that hematoma volume was smaller in NOAC-ICH compared to VKA-ICH,^9,10^ with better or similar functional outcome at hospital discharge.

We undertook an international, collaborative, multicenter, pooled individual patient data analysis to systematically describe the clinical and radiologic characteristics and outcome of NOAC-ICH in comparison to VKA-ICH.

## METHODS

We identified relevant cohorts from an international multicenter collaboration.^4^ To reduce bias and confounding due to secular trends in ICH treatment (including anticoagulation reversal strategies), we only included VKA-ICH data after the date of diagnosis of the first included NOAC-ICH at each center, according to a prespecified protocol. Inclusion criteria were as follows: ICH while on oral anticoagulation (NOAC-ICH or VKA-ICH); age over 18 years; for VKA-ICH, international normalized ratio (INR) on hospital admission ≥1.3; for NOAC-ICH, known NOAC use within 24 hours prior to ICH clinical symptoms. Exclusion criteria were as follows: secondary cause for ICH (such as major head trauma in the previous 24 hours, vascular malformations, tumors, cavernomas, aneurysms, other known coagulopathy, or hemorrhagic transformation of an infarct); or predominant subarachnoid hemorrhage. The primary outcome was mortality by 90 days, adjusted for potential confounding baseline characteristics. Secondary outcomes were ICH volume at baseline, proportion of patients with HE, and functional outcome measured by the modified Rankin Scale (mRS) at discharge. Some patient data from previously published studies^5,10^ were included (NOAC, n = 33; VKA, n = 52).

### Clinical and imaging data analysis.

We collected clinical, demographic, and imaging data using a standardized data collection form. Imaging data included baseline ICH volume measured from the first available CT scan, using either ABC/2 or semiautomated planimetric measurement, blinded to anticoagulant type and outcome in all but 3 centers; hematoma location (lobar, supratentorial deep [basal ganglia and thalamus], brainstem, or cerebellar); intraventricular hemorrhage (IVH) volume determined using a semiautomated planimetric method or the modified Graeb or Hallevi scales^4,11,12^; and HE, defined as an increase in volume by >33% or >6 mL from the baseline scan within 72 hours in patients with their first scan within 6 hours of symptom onset and without subsequent intracranial surgery before follow-up imaging, in keeping with a recent collaboration^4^ and validation.^13^

### Statistical analysis.

D.W., D.J.W., A.R.P.-J., and A.M. wrote a prespecified statistical analysis plan, which was approved by all participating centers. We compared patient characteristics between the VKA-ICH and NOAC-ICH groups using appropriate analysis for categorical and continuous variables. We visually inspected the distribution of the continuous variables using histograms, summarized as medians with interquartile range (IQR). Differences between the groups were determined using the Mann-Whitney test (if not normally distributed); categorical variables between the groups were compared with the χ^2^ test. We log-transformed variables that were not normally distributed. For IVH volume (where the majority of patients had 0 mL), we modeled using 2 variables: an indicator to model those with no IVH and a log-transformed variable for those with IVH, allowing us to generate a hazard ratio (HR) for each milliliter increase of IVH volume. Univariate Kaplan-Meier estimates were used to estimate survival probabilities by 90 days for each anticoagulant group; the log-rank test was used to compare groups.

Required sample sizes were estimated using a previously reported convenience sample.^4^ Assuming 90-day mortality (primary outcome) in the VKA group to be 46%, a study of 50 NOAC and 500 VKA patients has 80% power to identify a HR of 0.55 (i.e., mortality of 25% vs 46%) with a 2-sided *p* value of 0.05. For the secondary outcome of ICH volume, if we assume VKA-ICH median volume of 18 mL, a study of 50 NOAC and 500 VKA patients has 80% power to identify a difference of 61% (median 18 vs 11 mL) with a 2-sided *p* value of 0.05. For the secondary outcome of ICH expansion, we assume expansion (>6 mL or >33%) to occur in 60% of the VKA patients (and that only half of VKA and NOAC patients will have imaging at the required time windows); a study of 25 NOAC and 250 VKA patients has 75% power to identify a difference between 60% and 33% with a 2-sided *p* value of 0.05.

### Prespecified analyses.

For the primary prespecified outcome analysis, we fitted a Cox proportional hazards model, adjusting for variables known to affect outcome (age, sex, baseline Glasgow Coma Scale [GCS] score, log baseline ICH volume, baseline IVH volume, ICH location, and acute neurosurgery). The assumption of proportional hazards was assessed by visual inspection of each log–log plot of survival. Possible heterogeneity in general ICH management, resources, and ethnicity by site was addressed by adding a shared frailty term to the model. We performed prespecified subgroup analyses of the primary outcome measure by ICH location and by individual NOAC agent. A secondary analysis compared baseline ICH volume between anticoagulants using a linear regression model with log-transformed ICH volume as the dependent variable, adjusting for age and ICH location. HE (yes/no) between anticoagulant groups was investigated with logistic regression adjusting for age, ICH location, GCS, and baseline INR.

### Post hoc analyses.

We explored functional independence at discharge using the mRS and logistic regression. Discharge mRS was dichotomized into 0–2 vs 3–6 and adjusted for premorbid mRS, age, and baseline GCS. Shift analysis was the prespecified analysis of choice, but our data did not fulfil the proportional odds assumption. A second post hoc analysis was undertaken using data only within the first 30 days of follow-up. Furthermore, we ran individual sensitivity analyses of our primary outcome adjusting for (in addition to the pre specified variables) anticoagulation reversal with prothrombin complex concentrate (PCC) (yes/no), event to presentation time (days), adjustments for method of volume ascertainment (ABC/2 vs planimetric), and whether patients came from a consecutive registry. We also undertook further sensitivity analyses. First, we included a primary outcome of mortality by 30 days (which might be more directly related to the effect of the acute ICH); in this analysis, we included only age, sex, and acute neurosurgery in one model and age, sex, acute neurosurgery, ICH location, and IVH extension in another model, excluding baseline characteristics likely to be confounded by oral anticoagulant type (ICH volume, baseline GCS, IVH volume). Finally, we undertook a sensitivity analysis of mortality at 90 days to account for heterogeneity in the ICH volume measurement method.

### Standard protocol approvals, registrations, and patient consents.

This study was approved by the NHS Health Research Authority (Research Ethics Committee reference 15/YH/0475). Responsibility for ensuring compliance with local laws and data sharing policies was delegated to local principle investigators at each center, as outlined in the protocol.

## RESULTS

**Figure 1:**
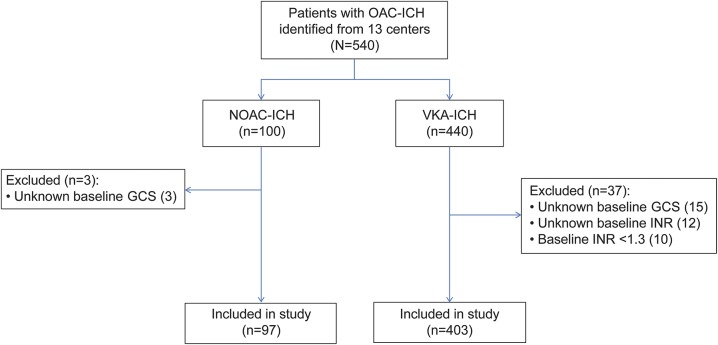
Flowchart of study design and patient selection GCS = Glasgow Coma Scale; ICH = intracerebral hemorrhage; INR = international normalized ratio; NOAC = non–vitamin K antagonist oral anticoagulant; OAC = oral anticoagulation; VKA = vitamin K antagonist anticoagulant.

We retrospectively pooled data from 13 stroke registries from the following countries: United Kingdom (2 centers and a multicenter prospective observational study), Finland, the Netherlands, Switzerland, France, Germany, Canada, Australia (2 centers), Taiwan, and Japan (table 1). We identified 540 patients with oral anticoagulant (OAC)–associated ICH recruited between April 28, 2012, and August 18, 2015 (registration periods varied by center); 100 participants had NOAC-ICH and 440 had VKA-ICH. We excluded 40 patients (figure 1); full datasets required for the primary analysis were available for 500 participants (97 with NOAC-ICH and 403 with VKA-ICH) (figure 1). The NOACs used were apixaban (n = 13), dabigatran (n = 13), and rivaroxaban (n = 69). Two participants were taking either rivaroxaban or apixaban. The median follow-up (accounting for deaths prior to 90 days) was 60 days (IQR 9–196) and varied by center (table 1). The onset of ICH symptoms to CT scan ranged from <24 hours (402 patients, 80%) to 11 days (1 patient, 0.2%). ICH volume was rated using the ABC/2 method in 8 centers and a semiautomated planimetric method in 5 centers. IVH was rated using the modified Graeb method in 3 centers, the Hallevi score in 6 centers, and a semiautomated planimetric method in 4 centers. The median INR value within the VKA-ICH group was 2.7 (IQR 2.2–3.4). The underlying indication for anticoagulation was known in 415/500 patients, as follows: 370 (89%) for AF, 28 (6.7%) for deep vein thrombosis/pulmonary embolism, 15 (3.6%) for mechanical heart valves, and 2 (0.5%) for cardiomyopathy. All but 1 of these patients was on a VKA.

**Table 1:**
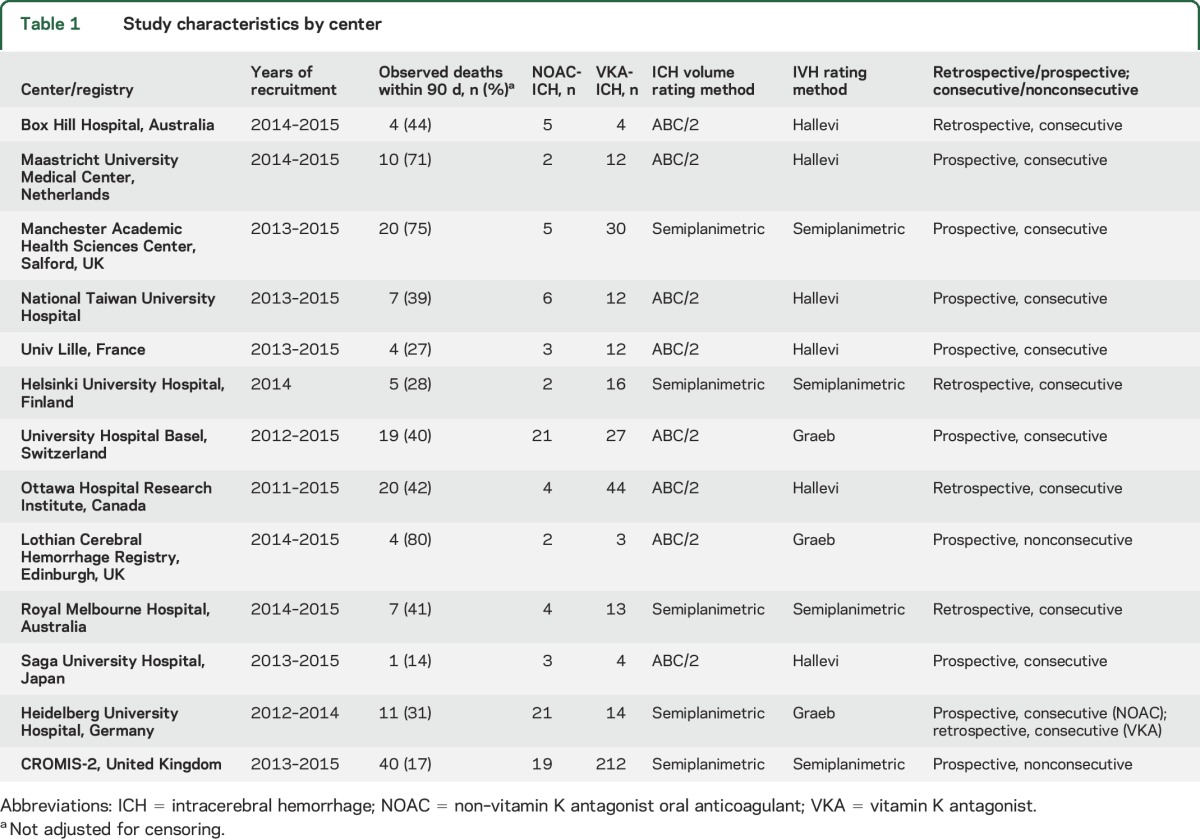
Study characteristics by center

The patient characteristics for NOAC-ICH and VKA-ICH are shown in table 2; those with NOAC-ICH more often had cerebellar ICH and worse premorbid mRS compared with VKA-ICH, but were otherwise similar. OAC reversal information was available in 450 patients (365 VKA-ICH and 85 NOAC-ICH); of these, 286/365 (78%) participants with VKA-ICH received 3 or 4 factor PCC, compared to 35/85 (41%) of participants with NOAC-ICH (*p* ≤ 0.001). Twenty-four patients with VKA-ICH (6%) and 7 patients with NOAC-ICH (7%) were treated with acute neurosurgery.

**Table 2:**
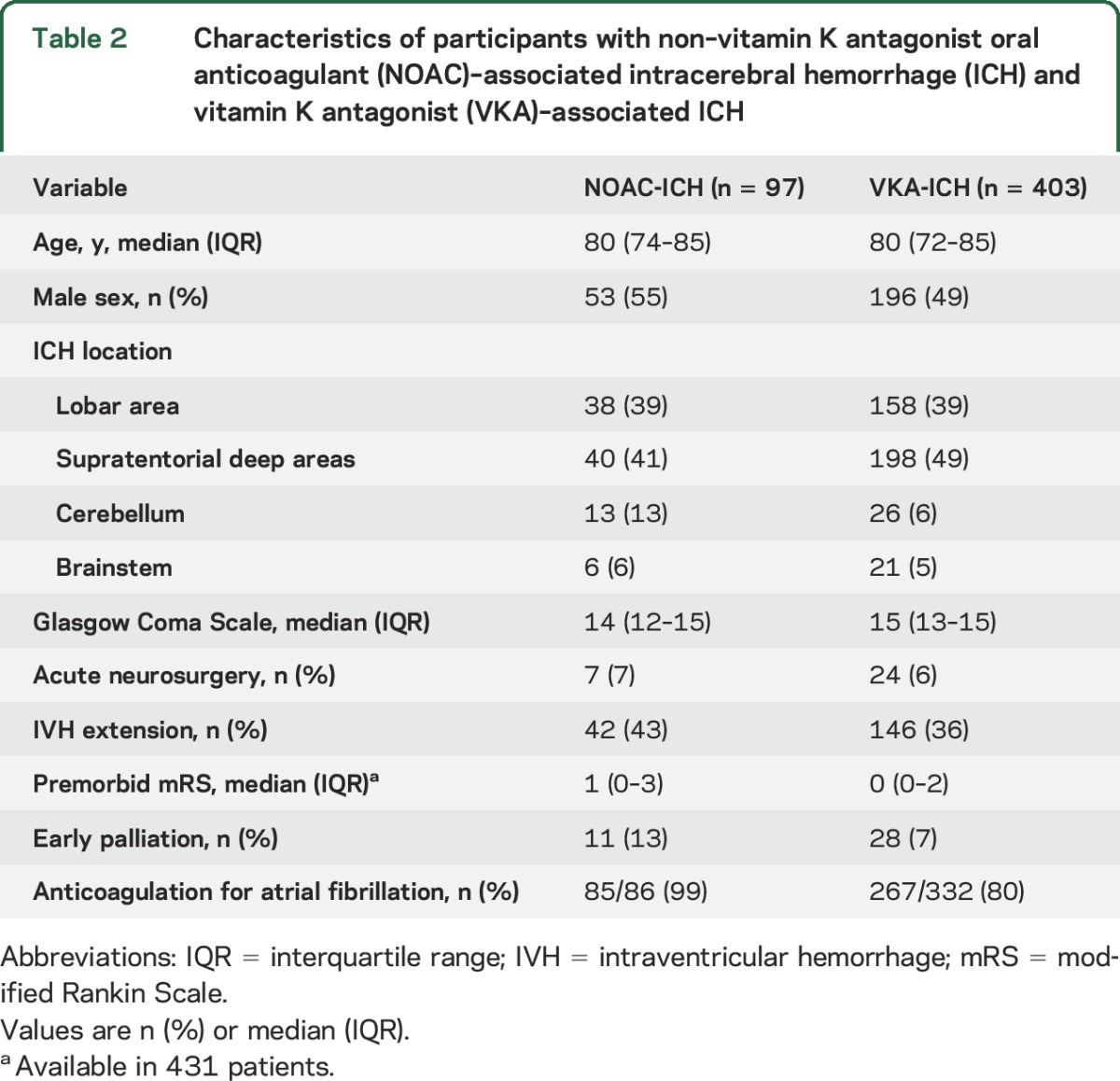
Characteristics of participants with non–vitamin K antagonist oral anticoagulant (NOAC)–associated intracerebral hemorrhage (ICH) and vitamin K antagonist (VKA)–associated ICH

### Primary outcome.

**Figure 2:**
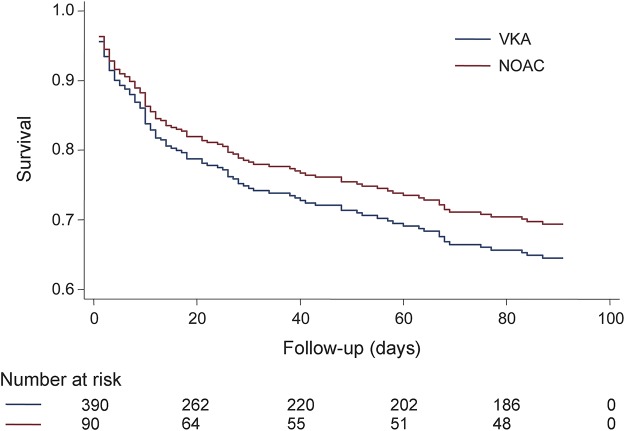
Survival curve comparing non–vitamin K oral antagonist anticoagulant (NOAC)–associated intracerebral hemorrhage (ICH) and vitamin K antagonist anticoagulant (VKA)–associated ICH 90-day mortality Adjusted for age; sex; baseline Glasgow Coma Scale score, ICH location, and log volume; intraventricular hemorrhage volume; and intracranial surgery.

There were 161 deaths (a crude mortality rate of 32%). After adjusting for censoring, all-cause mortality was 31% at 90 days. Ninety-day mortality was 33% (95% confidence interval [CI] 24–44) for NOAC-ICH vs 31% (95% CI 27–37) for VKA-ICH (*p* = 0.64). Age, admission GCS, and ICH volume were associated with 90-day mortality in univariable analysis (table 3). After adjusting for baseline variables (age, sex, baseline GCS, log baseline ICH volume, baseline IVH volume, ICH location, and acute neurosurgery) and addressing heterogeneity by site (using a shared frailty term), there was no difference in survival between NOAC-ICH and VKA-ICH (HR 0.93; 95% CI 0.52–1.64; *p* = 0.79) (figure 2 and table 3). In post hoc analyses, the primary result was unchanged as follows: PCC treatment (HR 0.99; 0.57–1.72), ICH onset to presentation time (HR 0.81; 95% CI 0.49–1.35), ICH volume measurement method (HR 0.80; 95% CI 0.48–1.35), and consecutive registry source (HR 0.84; 95% CI 0.50–1.38). There was no between-group difference in 30-day mortality (HR NOAC-ICH vs VKA-ICH 0.83; 95% CI 0.47–1.48; *p* = 0.53) or 60-day mortality (HR NOAC-ICH vs VKA-ICH 0.75; 95% CI 0.44–1.30; *p* = 0.308).

**Table 3:**
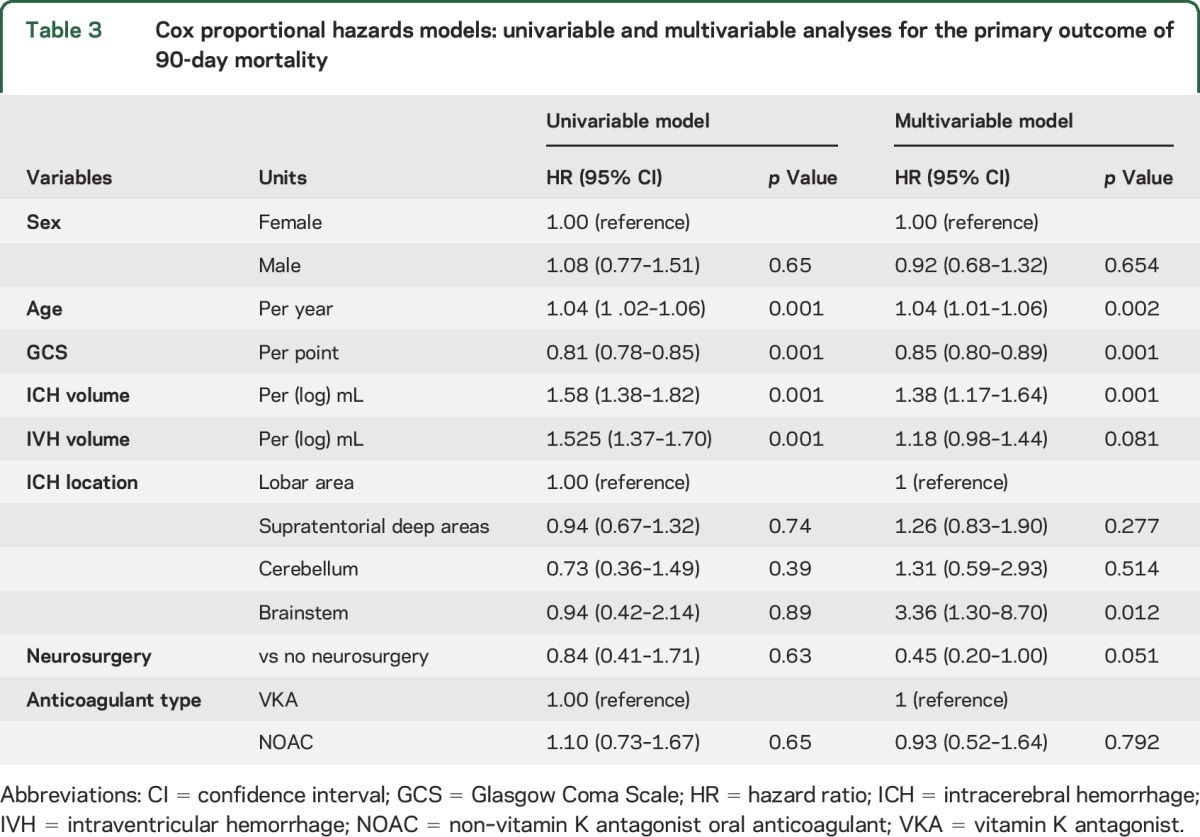
Cox proportional hazards models: univariable and multivariable analyses for the primary outcome of 90-day mortality

For the outcome of mortality by 30 days (including age, sex, acute neurosurgery in model 1 and age, sex, acute neurosurgery, ICH location, and IVH extension as covariates in model 2) the main result was unchanged (HR for NOAC-ICH vs VKA-ICH 0.88 [95% CI 0.53–1.45] for model 1 and 0.94 [95% CI 0.55–1.59] [*p* = 0.813] for model 2) (supplemental data at Neurology.org). In sensitivity analyses for the outcome of mortality by 90 days including log ICH volume assessed by different methods (ABC/3, planimetry), the main result for NOAC-ICH vs VKA-ICH also remained unchanged (see supplemental data).

### Secondary outcomes.

Median baseline VKA-ICH volume was 10.6 mL (IQR 4.0–27.9), compared to 14.4 mL (IQR 3.6–38.4) for NOAC-ICH (*p* = 0.78). There was no statistical difference in log-transformed ICH volumes in multivariable linear regression (coefficient 0.06; 95% CI −0.28 to 0.41, *p* = 0.72). Data regarding HE were available in 188 patients (140 [35%] VKA-ICH and 48 [49%] NOAC-ICH), and did not vary by anticoagulant type (NOAC-ICH 29/48 40% vs VKA-ICH 93/140 34%, *p* = 0.45); there was no between-group difference in HE after multivariable logistic regression adjusting for age, ICH location, baseline INR, and GCS (OR 1.38; 95% CI 0.46–4.18; *p* = 0.57).

Participants with VKA-ICH were more likely to be functionally independent at discharge than those with NOAC-ICH in univariable analysis (23% vs 10%, respectively, *p* < 0.001), but not in multivariable logistic regression after adjusting for premorbid mRS, age, IVH extension, and baseline GCS (NOAC vs VKA-ICH: OR 0.47; 95% CI 0.18–1.19; *p* = 0.11). Backwards stepwise regression suggested that a baseline difference in premorbid mRS between NOAC-ICH and VKA-ICH accounts for the difference between the univariable and multivariable analysis results.

## DISCUSSION

Our multicenter, international individual patient pooled analysis of 500 participants with OAC-ICH found that 90-day mortality for NOAC-ICH is similar to that for VKA-ICH. Functional outcome, ICH volume, and HE were also similar between the NOAC-ICH and VKA-ICH groups. We found no statistically significant differences between individual NOAC-ICH 90-day mortality rates, although the numbers of participants taking each individual OAC were limited. We found no significant difference between NOAC-ICH and VKA-ICH in a post hoc analysis of 30-day mortality.

Although previous small studies suggested that hematoma volume was smaller for NOAC-ICH compared to VKA-ICH, with better or similar functional outcome at hospital discharge, these included only 5 and 11 participants with NOAC-ICH, respectively,^9,10^ and were not statistically powered to detect differences in ICH volume or outcome. Furthermore, hospital discharge mRS might not reflect longer-term outcome and is prone to confounding by premorbid mRS. We included 90-day mortality, which more accurately reflects longer term outcome and is less prone to confounding. Although previous animal models suggest that hematoma expansion does not occur with therapeutic doses of NOACs (rivaroxaban and dabigatran),^14,15^ supported by small human case series (n = 5 and 6),^9,16^ a recent multicenter registry (n = 61) described hematoma expansion with NOACs,^5^ consistent with our study. The real-world mortality we report for NOAC-ICH and VKA-ICH is in keeping with findings from the RE-LY (mortality for VKA-ICH 36% vs dabigatran-ICH 35% [150 mg] and 41% [110 mg]),^6,17^ ROCKET-AF (mortality for VKA-ICH 50% vs rivaroxaban-ICH 48%),^7^ and ARISTOTLE (mortality for VKA-ICH 42.3% vs apixaban-ICH 45.3%)^8^ randomized trials.

Our study has important strengths. We include a large cohort of patients with both NOAC-ICH and VKA-ICH. The use of detailed individual patient clinical and radiologic data allowed us to describe baseline characteristics and key clinically relevant outcomes beyond the acute phase of hospital admission, and to adjust for important potential confounding factors. Because we included prospectively collected clinical data from Europe, North America, East Asia, and Australasia, our findings are likely to be more generalizable to a real-life OAC-ICH population in broad range of health care settings than any of the previous studies. We reduced bias from secular treatment trends by only including NOAC-ICH and VKA-ICH recruited during the same time period, and from the same patient population, from each participating center. Our primary outcome of 90-day mortality is unlikely to be substantially confounded by premorbid functional status.

We also acknowledge limitations: functional outcome at 90 days would have been a preferable secondary outcome to discharge mRS, as it is less confounded by length of stay, but these data were not available. We do not know how widely NOAC adoption was within each source population, which could lead to confounding by indication. Although we compared and adjusted for baseline characteristics between NOAC-ICH and VKA-ICH patients, there might be unmeasured confounding factors, including clinician judgement used in choosing between a VKA or a NOAC, alcohol intake, blood pressure variability, or comorbidities such as renal disease, which we could not fully account for. These may be of some concern especially as NOACs are contraindicated in some settings. Two registries did not enroll every patient with OAC-ICH; exclusion of severe ICH might have caused ascertainment bias, but in our post hoc sensitivity analysis this did not affect 90-day mortality.

Our reported 90-day mortality (31%) is lower than some previous series of anticoagulant-related ICH (range 46%–68%)^18^ although similar to that reported in a recent German multicenter registry study (28%).^5^ Follow-up duration and ICH mortality varied between centers; although we used a shared frailty model, this cannot fully account for all between-center heterogeneity. We acknowledge some measurement heterogeneity due to 2 different methods for rating ICH volumes, but ensured that each center rated both VKA-ICH and NOAC-ICH using the same method. Furthermore, adjusting for this heterogeneity in sensitivity analysis did not alter the primary result. Finally, hematoma expansion data were only available in 188/500 patients, and were more often available in NOAC-ICH, so might not be generalizable to the entire cohort.

Our finding that ICH volume, HE, and functional outcomes were similar for NOAC-ICH and VKA-ICH might have implications for clinical practice. NOACs have a lower risk of ICH than VKA for ischemic stroke prevention in populations with AF.^1^ Reversal agents for NOAC-related hemorrhage, including NOAC-ICH, have been developed, and are likely to become more widely available (although these agents are not yet established as clinically effective in patients with NOAC-ICH).^19–22^ Our study therefore does not support previous concerns that NOAC-ICH might have poorer outcome than VKA-ICH because of a lack of available specific reversal agents.^2,3^ Nevertheless, further randomized data for detailed NOAC-ICH functional outcome and radiologic profiles compared to VKA-ICH are needed. Furthermore, little is known about the risk of NOAC-ICH or its functional outcome in patients at high risk of ICH, e.g., those with previous ICH or with bleeding-prone arteriopathies.^23^ As specific NOAC-ICH reversal agents become available, studies of how these affect clinical and radiologic outcomes are needed. Other treatment options for NOAC-ICH, including hemostatic agents^24^ and acute blood pressure management,^25^ also need to be investigated, ideally in randomized controlled trials.

## GLOSSARY

AF: atrial fibrillation
CI: confidence interval
GCS: Glasgow Coma Scale
HE: hematoma expansion
HR: hazard ratio
ICH: intracerebral hemorrhage
INR: international normalized ratio
IQR: interquartile range
IVH: intraventricular hemorrhage
mRS: modified Rankin Scale
NOAC: non–vitamin K antagonist oral anticoagulant
OAC: oral anticoagulant
PCC: prothrombin complex concentrate
VKA: vitamin K antagonist
